# Clinical and Novel Biomarkers in Penile Carcinoma: A Prospective Review

**DOI:** 10.3390/jpm12091364

**Published:** 2022-08-24

**Authors:** Leonel Pekarek, Miguel A. Ortega, Oscar Fraile-Martinez, Cielo García-Montero, Carlos Casanova, Miguel A. Saez, Natalio García-Honduvilla, Melchor Alvarez-Mon, Julia Buján, Victor Diez-Nicolas, Javier F. Burgos, Victoria Gomez Dos Santos

**Affiliations:** 1 Department of Medicine and Medical Specialities, Faculty of Medicine and Health Sciences, University of Alcalá, 28801 Alcala de Henares, Spain; 2 Ramón y Cajal Institute of Sanitary Research (IRYCIS), 28034 Madrid, Spain; 3 Oncology Service, Guadalajara University Hospital, 19002 Guadalajara, Spain; 4 Cancer Registry and Pathology Department, Principe de Asturias University Hospital, 28806 Alcala de Henares, Spain; 5 Pathological Anatomy Service, Central University Hospital of Defence-UAH Madrid, 28801 Alcala de Henares, Spain; 6 Immune System Diseases-Rheumatology, Oncology Service an Internal Medicine (CIBEREHD), University Hospital Príncipe de Asturias, 28806 Alcala de Henares, Spain; 7 Department of Surgery, Medical and Social Sciences, Faculty of Medicine and Health Sciences, University of Alcalá, 28801 Alcala de Henares, Spain; 8 Urology Department, Ramon y Cajal Hospital, Alcala University, IRYCIS, 28034 Madrid, Spain

**Keywords:** penile cancer, serological markers, histological markers, microRNA

## Abstract

Penile carcinoma is a rare urological neoplasia in men compared to other more common tumors, such as prostate, kidney, or bladder tumors. However, this neoplasm continues to affect a large number of patients worldwide, with developing countries presenting the highest incidence and mortality rates. Important risk factors such as the human papilloma virus, a factor affecting a large number of patients, have been described; however, few studies have evaluated screening programs in populations at risk for this disease, which severely affects the quality of life of older men. The management of these patients is usually complex, requiring surgical interventions that are not without risk and that have a great impact on the functionality of the male reproductive system. In addition, in cases of disseminated disease or with significant locoregional involvement, patients are evaluated by multidisciplinary oncological committees that can adjust the application of aggressive neoadjuvant or adjuvant chemotherapy on numerous occasions without clear improvement in survival. Chemotherapy regimens are usually aggressive, and unlike in other urological neoplasms, few advances have been made in the use of immunotherapy in these patients. The study of serological and histological biomarkers may help to better understand the underlying pathophysiology of these tumors and select patients who have a higher risk of metastatic progression. Similarly, the analysis of molecular markers will improve the availability of targeted therapies for the management of patients with disseminated disease that would benefit prognosis. Therefore, the purpose of this article is to summarize the main advances that have occurred in the development of serological and histological markers and their therapeutic implications in patients diagnosed with penile carcinoma, explaining the limitations that have been observed and analyzing future perspectives in the management of this disease.

## 1. Introduction

Penile cancer is a rare entity that usually presents as an asymptomatic lesion or ulcer on the penis that is sometimes present for a long period of time. The vast majority of malignant neoplasms of the penis correspond to squamous epithelial carcinoma. At the epidemiological level, this neoplasm is very rare in developed countries, where it represents less than 1% of malignant neoplasms in men and has an incidence of approximately 1.33 cases per 100,000 men in Europe [1]. In Spain, the incidence is approximately 1.5 cases per 100,000 men. This is not so in developing countries, where penile carcinoma accounts for up to 20% of malignant neoplasms in men [2,3,4]. This disease usually affects older men, and the average age of diagnosis is approximately 60 years. The disease also affects more Hispanic people than people of other ethnicities. Numerous risk factors, especially phimosis and human papillomavirus infection (HPV), have been described. Phimosis is associated with worse genital hygiene, which leads to the accumulation of smegma, which promotes a situation of chronic inflammation that causes dysplastic changes in the epithelium and subsequently the appearance of a true invasive neoplasia. This is why up to 90% of penile cancers are associated with phimosis and situations of poor hygiene, such as severe obesity or genital trauma [5]. Notably, circumcision reduces the risk of infection by sexually transmitted diseases and oncogenic variants of the human papillomavirus. HPV, which has also been associated with cervical cancer and anal cancer, is found in approximately half of patients with penile cancer and usually corresponds to the 16 variants that represent up to 68% of HPV infections [6]. It is noteworthy that, as in patients with oropharyngeal carcinoma, the presence of human papillomavirus has been described as a good prognostic factor through the activation of E6 and E7 oncogenes, which inhibits cell cycle control proteins such as p53 or pRb [7]. In the study conducted by Djajadiningrat et al., it was found that in 212 patients with penile carcinoma, survival at 5 years was higher for patients who were human papillomavirus positive than for those were negative [8]. Relatedly, in a meta-analysis of 20 studies, Freja Lærke Sand et al. found that positivity for p16, which is an immunohistochemical marker of HPV, is associated with a better prognosis. Currently, there are no screening programs for this disease [9]. This may be because developed countries have a low incidence, but in regions where a large part of the population is at risk, screening has not been studied with urological examinations, serum markers, or any other technique, unlike for cervical cancer in women where HPV detection presents a specific diagnostic and management algorithm [10].

From an anatomopathological point of view, the vast majority of malignant lesions (>95%) of the penis correspond to squamous cell carcinomas. The other 5% of lesions usually correspond to rare neoplasms such as Kaposi sarcoma, cutaneous lymphomas, or different types of sarcomas, where a situation of severe immunodeficiency is usually present. The precursor lesion of penile cancer is penile intraepithelial neoplasia (PeIN). In this lesion, epithelial dysplastic changes are observed without alteration of the basement membrane; a combination of entities are grouped together here, such as erythroplasia of Queyrat or Bowen’s disease, where there are specific anatomopathological characteristics that share dysplastic alterations in the squamous epithelium [11,12]. It is important to note that the risk of PeIN progression to squamous carcinoma itself is 7–8% in those lesions defined as severe or PeIN III [13]. At the diagnostic level and following the classification of the College of American Pathologists, the subtypes of invasive squamous lesions can be differentiated by their relationship with human papillomavirus. For example, condylomatous, basaloid, and verrucous histology are the histologies that are most associated with HPV, while the usual papillary or sarcomatoid expression is not usually related to histological changes caused by HPV. In the same way, the diagnosis usually includes the degree of differentiation of the tumor and the locoregional extension [14].

The differential diagnosis includes a large number of inflammatory and infectious entities, such as genital psoriasis, angiokeratomas, lichen planus, genital herpes infection, or syphilis, as well as the previously described premalignant lesions. In most cases, a certain diagnosis is established by biopsy, which also allows the study of HPV expression [15]. The diagnostic complexity of this disease lies in the difficulty of establishing locoregional or disseminated involvement. For example, in the case of suspected invasion of corpora cavernosa or other local structures, the preferred imaging method is magnetic resonance imaging over computerized axial tomography [16]. If there is a high probability of inguinal lymphatic dissemination, patients can undergo studies such as dynamic sentinel lymph node biopsy, which has a reported sensitivity of up to 88% in demonstrating lymphatic invasion or lymphatic superficial inguinal dissection, although it is accompanied by a higher complication rate than the previous procedure [17,18]. If a large adenopathy is observed in the physical examination, a lymph node biopsy can also be performed. In any case, when there is suspicion of lymphatic invasion and a subsequent disseminated disease, a complete imaging study, such as thoracic pelvic axial computed tomography, should be performed to study metastatic disease [19]. Normally, distant extension does not require a brain imaging test since metastatic brain dissemination is rare in squamous cell carcinoma of the penis [20]. Regarding the treatment of penile carcinoma, it is usually individualized and agreed upon in multidisciplinary tumor committees where different experts, both oncologists and urologists, evaluate the patient as a whole to decide the best therapeutic option. In the initial stages where tumors are localized, limited excision or noninvasive therapies (such as topical therapy with fluorouracil and imiquimod, Mohs micrographic surgery, or radiotherapy) can be used to preserve both the anatomy and the functionality of the penis [21,22]. The 10-year survival rate of patients with local tumors without lymphatic extension who are treated with curative surgery is 96%, while lymphatic invasion decreases 10-year survival to 50% or, if there is metastatic extension, 12% [23,24]. In tumors with more aggressive anatomopathological characteristics, such as lymphovascular or neural invasion accompanied by large lesions, partial or total amputation is usually the initial treatment. The most frequent intervention is partial resection, which occurs in the majority of surgical interventions for tumors with a high risk of progression [25]. It should be noted that in T1a lesions where there is subepithelial invasion, the rate of lymphatic invasion is up to 18%. In the case of T1b where there is invasion of the underlying connective tissue, the presence of lymphatic metastases is evident in up to 50% of patients. In cases where there is perineural or lymphovascular invasion, the lymphatic dissemination rate can be up to 80%, which substantially decreases the prognosis of these patients [26,27,28]. In those patients where an unresectable tumor, bilateral inguinal invasion, or pelvic lymphatic invasion is observed, patients are subjected to neoadjuvant chemotherapy with paclitaxel, ifosfamide, and cisplatin. Likewise, the same chemotherapy regimen can be used as an adjuvant associated with radiotherapy. It should be noted that up to 30% of patients have both locoregional and distant recurrence with a time interval of distant lesions of up to 10.5 months [29,30]. On the other hand, patients with disseminated metastatic disease at diagnosis can be subjected to a chemotherapy regimen of paclitaxel, ifosfamide, and cisplatin with response rates of up to 38% [31]. We cannot forget that the use of immunotherapy in these patients is being evaluated in different clinical trials, both in monotherapy and associated with radiotherapy (NCT03686332 or NCT04224740), but it is not currently available in daily clinical practice, unlike for other urological neoplasms. For this reason, in recent years, the histological study of penile carcinoma has gained importance in the detection of the presence of HPV. Therefore, we are faced with a rare urological neoplasm that is not well studied and has limited therapeutic options in advanced cases. We cannot forget that despite being a forgotten oncological disease, the study of the underlying pathophysiology of these tumors can help us understand the complexity of metastatic disease and design therapies or early diagnostic methods in patients at high risk of tumor progression and dissemination, which will allow us to better understand this disease.

## 2. Histological Markers

Unlike other tumors where the importance of histological markers by immunohistochemical techniques has been revealed, allowing the standard of care to be improved, the same has not happened in penile carcinoma. The study of histological molecular markers represents a significant advance in breast, lung, colon, and ovarian cancer, which is something that has not happened in penile carcinoma [32]. The importance of molecular alterations with tissue expression has allowed the development of targeted therapies and the study of chemoradiotherapy regimens to offer an improvement in survival. The usefulness of the immunohistochemical marker p16 as a prognostic factor has been demonstrated in penile carcinoma [33]. It should be noted that p16 is a protein encoded by the tumor suppressor gene CDKN2A, which is an inhibitor of cyclin-dependent kinases that act by regulating the cell cycle in G1-S by inactivating the protein encoded by the retinoblastoma gene (pRb). Different authors have shown that in the pathogenesis of cervical or oropharyngeal cancer, the integration of the viral genetic material in the genome of the host cells and the consequent expression of the products of the viral genes E6 and E7 cause the inactivation of both p53 and pRb [34]. This inactivation therefore leads to overexpression of p16, which can be detected by immunohistochemical techniques. Therefore, the expression of p16 is correlated with the integration of the viral genome into the host genome in cells. We must highlight how numerous authors have described the prognostic importance of p16 in pharyngeal carcinoma; p16 is currently considered the strongest independent prognostic factor in these tumors and is associated with better survival in patients with high expression than in patients with low expression of p16 [35]. Along these lines, different authors have demonstrated the usefulness of p16 in penile carcinoma as a prognostic factor. For example, the UpToDate authors Djajadiningrat et al. evaluated 212 patients with penile carcinoma and found that survival at 5 years was significantly higher in patients with elevated p16 than in those without p16 elevation (5-year overall survival of 96% versus 82%) [8]. In a meta-analysis that included 323 patients with penile cancer from different studies, Zhang et al. observed how the expression of p16 is accompanied in effect by an improvement in survival [36]. We must emphasize the importance of these findings since their determination through a simple and easy-to-interpret technique, namely, immunohistochemistry, has allowed us to anticipate the prognosis of these patients. Likewise, other authors have studied different histological markers in relation to prognostic factors. For example, in 28 patients, Protzel et al. showed how high immunohistochemical ki67 is accompanied by a worse prognosis and greater lymphatic invasion than low expression [37]. Likewise, Panic et al. showed that elevated caveolin 1 expression in the tumor tissue of 43 patients with penile carcinomas was associated with a worse prognosis [38]. Relatedly, Gunia et al. reported the expression of p53, p21, and cyclin D1 in 11 patients with penile carcinoma with elevated levels of p53 could be used as a prognostic factor; however, in a multivariate analysis, the relationship for p21 or cyclin d1 was not strong enough for them to be used as prognostic markers [39]. These results agree with those of Prapiska et al., where they observed in 33 patients with penile carcinoma that the 3-year survival was 18% in those patients with high p53 expression, while it was 60% in those with low p53 expression, demonstrating its utility as a prognostic factor [40]. It should be noted that numerous authors have studied new markers based on their relationship with HPV infection. For example, Chaux et al. studied the immunohistochemical expression of biomarkers related to mTOR cini PTEN, phosphoAKT, phosphomTOR, and phospho-S6 in 112 patients with penile carcinoma, where there is deregulation of these markers regardless of HPV infection, and observed their usefulness as prognostic factors [41]. The discovery of PDL1 expression in lung or breast cancer has been a real revolution in terms of the monoclonal antibody-based targeted therapies developed since. Therefore, different authors have studied the expression of PDL1 in penile carcinoma. For example, Davidsson et al. observed elevated levels of PDL1 and microsatellite instability with alterations in MLH1, PMS2, MSH2, and MSH6 measured by immunohistochemistry in 222 men with penile carcinoma. In those patients with elevated PDL1 expression, the median survival was 1.5 years, whislt in those patients with negative expression, the median survival was 3.12 years; this is in addition to the fact that elevated levels were accompanied by more aggressive tumor behavior, demonstrating the usefulness of PDL1 as a prognostic factor [42]. Although there have been case reports of metastatic disease in patients with penile carcinoma, several clinical trials are being carried out to evaluate the response to therapy directed against PDL1; in particular, NCT04224740 aims to evaluate the combination of cisplatin with pembrolizumab in patients with metastatic penile carcinoma. Therefore, we can observe how the study of immunohistochemical markers allows the design of possible targeted therapies that should be evaluated and those that are currently being evaluated in different clinical trials.

On the other hand, we cannot forget the processes of angiogenesis and lymphangiogenesis. Understanding these processes has allowed us to better understand the microcirculation and vascular invasion of different tumors, and thus, to not only study the mechanism of lymphatic dissemination and metastasis but also adequately evaluate the risk of invasion of these tumors. Currently, the CAP criteria evaluate the lymphatic, vascular, and neural invasion of penile carcinoma samples to establish the correct anatomopathological stage of the tumor. The concept of vascular microdensity represents a measure of the number of blood vessels per wide-magnification field under the microscope [43]. In this way, a high density of microvessels cancers promotes tumor growth, worsens the prognosis, and, in cases of oxygen and nutrient depletion, enables necrosis in urological tumors such as kidney, prostate, or bladder cancer [44]. Similarly, the importance of vessel microdensity in penile cancer has been studied. For example, in an immunohistochemistry evaluation of CD34 expression in 64 patients with carcinoma of the penis, AmrAl-Najar have demonstrated that patients with a high and low density of microvessels have 5-year survival rates of 75% and 30%, respectively [45]. It should be noted that the study of microvessel density is complex given that there are discrepancies with respect to these results. For example, Arora et al. evaluated the microdensity of vessels in 226 patients, where the 5-year survival of patients with low MDV was 79%, whilst that of patients with high MDV was 39% [46]. This may be due to different reasons, such as evaluations of the disease at different stages, lack of analysis of the entire neoplasia, or the specific immunohistochemical markers chosen. For example, CD34 (a marker of angiogenesis) may be elevated in fibroblasts of the tumor stroma and is present in both neovessels and nonfunctioning vessels [47]. Therefore, the study of angiogenesis markers does not currently allow the use of this marker as a prognostic factor given the discrepancies between different authors. On the other hand, lymphatic invasion has also been studied by different authors, and the main mechanism of metastasis of these tumors is through the lymphatic route. Minardi et al. evaluated the expression of podoplanin or D2-40 in 39 patients, observing after the analysis of ROC curves that an elevated intratumoral lymphatic density greater than 2 had a sensitivity of 83.3% and a specificity of 78% in predicting lymphatic metastatic invasion. When performing a multivariate analysis, there were no differences in mortality as a function of the level of expression of D2-40, which limits its use as a prognostic factor in penile cancer [48]. Therefore, the study of immunohistochemical markers allows us to complement the histological study by either allowing better stratification of those patients with greater probability of lymphatic invasion or, in the case of markers that can be used as prognostic factors, to establish a stricter follow-up routine in addition to being able to design possible targeted therapies that provide a real revolution in the management of urological neoplasms.

## 3. Serological Markers

Serological markers in oncology represent a set of proteins or molecules detectable in liquid biopsy (usually peripheral blood) that allow us to evaluate a patient with suspected neoplasia in an integral way. For example, these markers can be used in the screening, diagnosis, and follow-up of these patients where elevations in these markers represent a greater tumor burden or tumor recurrence, which allows us to shorten an otherwise long-term follow-up. Numerous serological markers are used in daily clinical practice. For example, CA 19-9 is useful in pancreatic cancer, and Ca-125 in ovarian cancer for the monitoring of high-risk women [49,50]. In urological neoplasms, urine cytology is currently used in bladder cancer, but there are no markers in real clinical practice that are used for penile carcinoma [51]. The importance of the detection of HPV in these tumors has been studied, but there are currently no screening programs for high-risk populations where serological markers are used in the disease [52]. It should be noted that numerous markers have been studied. In particular, it is worth highlighting the study by Ghoshal et al., where the expression of cellular protein, albumin, and haptoglobin was studied in 205,717 patients for 20 years to assess the usefulness of these markers in evaluating the risk of developing cancer of the penis or testicle; no significant associations were found [53]. When studying C-reactive protein levels in 51 patients with penile carcinoma undergoing curative surgery in different stages, Ghazal et al. observed how the preoperative increase in CRP is accompanied by lymphovascular invasion, which allows the prediction of lymphatic metastasis in these patients [54]. Likewise, Mo et al. studied the marker CCL20 (chemokine CC ligands) in 76 patients with penile carcinoma, demonstrating that preoperative serological levels were elevated compared to those of healthy controls, with a ROC curve value of 0.855, a sensitivity of 72.4%, and a specificity of 93.5%. In a comparison of penile carcinoma patients with healthy controls, it was also shown that serological elevation of CCL20 has a worse prognosis [55]. Other authors have studied less-common markers, such as laminin gamma 2 (LAMC2). Zhou et al. evaluated 114 patients with penile carcinoma and found that the expression and serological elevation of LAMC2 is accompanied by a higher lymphatic metastasis and is associated with worse survival than lower serological levels [56]. One of the main limitations of the use of tumor serological markers in penile carcinoma is that they require the presence of advanced lymphatic/vascular invasion, which occurs in advanced lesions. In addition, at the screening level, a genital physical examination would be much more efficient for the screening of this disease in high-risk populations. Therefore, unlike other tumors, the use of serological tumor markers in patients with penile carcinoma has not been fully studied, nor have adequate screening programs been proposed in high-risk populations such as certain populations in South America.

## 4. MicroRNA

In recent years, the usefulness of microRNAs as biomarkers has been appreciated not only in understanding the pathophysiology of the underlying disease, but also in the diagnosis and screening of different oncological diseases. MicroRNAs are small noncoding RNA molecules of approximately 20 nucleotides that regulate posttranscriptional genes that are related to the processes of cellular differentiation, proliferation, and apoptosis and promote or suppress the expression of target genes after transcription. A microRNA molecule posttranscriptionally regulates up to 200 different genes, and its study allows us to understand the underlying pathophysiology of the metastatic process [57]. The involvement of microRNAs in penile cancer has been described by different authors and is related not only to tumor proliferation or lymphovascular invasion, but also to a wide variety of molecular pathways, such as the Wnt, MAPK, p53, PI3K-Akt, Notch, and TGF-β pathways, allowing a better understanding of the mechanisms of penile carcinoma invasion [58,59,60]. In recent years, the importance of microRNAs has been studied by very few authors. For example, Ayoubian et al. described the expression of different microRNAs as a function of the expression of HPV in 47 subjects (27 patients with penile carcinoma vs. 18 healthy controls), demonstrating that there are 876 microRNAs that are both downregulated and upregulated, depending on HPV positivity. These changes in microRNA expression also occurred in healthy HPV-positive controls. It should be noted how the alterations in miR-211-5p and miR-181d-5p correspond to malignant lesions associated with HPV, including basaloid and verrucous carcinomas, among others. In addition, underexpression of miR-137 was observed, and miR-328-3p was more characteristic of patients with metastatic disease. On the other hand, we have seen the importance of targeted therapies in relation to PDL1expression. In this cohort, 48% of patients had overexpression of PDL1, and upregulation of miR-138-5p has been related to the expression levels of PDL1 in these patients [61]. Other authors, such as Hartz et al., evaluated 24 patients with penile carcinoma to determine how the underexpression of miR-1, miR-101, and miR-204 was associated with lymphovascular and metastatic invasion in addition to a worse prognosis in those patients, and the results demonstrated the prognostic utility of these biomarkers [62]. Pinho et al. evaluated the diagnostic and prognostic utility of miR-223-3p, miR-107, and miR-21-5p in 50 patients with penile carcinoma, demonstrating that these microRNAs were more frequent in neoplastic lesions than in adjacent nonneoplastic tissue and that patients with overexpression of miR-223-3p had a worse prognosis associated with a greater predisposition to lymphovascular invasion. The same occurred with the upregulation of miR-107, with patients exhibiting a lower average survival time than patients with lower expression levels. These microRNAs are associated with a decrease in the activity of the tumor suppressor enzyme PTEN in addition to alterations in the mitogen-activated protein kinase pathway MAPK (ERK1/ERK2), which is associated with cell proliferation and mechanisms of lymphovascular invasion [63]. As we have seen previously, the anatomopathological diagnostic criteria of penile carcinoma takes into account perineural invasion. Therefore, Pinho et al. observed the association between miR-145 downregulation and perineural invasion in 52 patients diagnosed with penile carcinoma related to alterations in oncogenes such as EGFR or C-MYC [64]. In relation to the diagnostic utility of differentiating neoplastic from non-neoplastic lesions, one of the most interesting studies carried out was that by Kausne et al., who examined 23 patients with penile carcinoma, analyzing up to 81 microRNAs. Their results allowed us to obtain ROC curves for miR-31-5p, miR-224-5p, and miR-223-3p with areas under the curve of 0.861, 0.739, and 0.733, respectively, demonstrating their usefulness for diagnosis [65]. We cannot forget the study by Zhang et al., who, using next generation sequencing (NGS), observed how up to 30 microRNAs were downregulated and 26 upregulated. This analysis supports the results of other authors, such as Ayoubian et al., who also observed the downregulation of miR-211-5p and miR-328-3, and Hartz et al., who reported the downregulation of miR-204 [66]. Therefore, we can observe the usefulness of microRNAs as biomarkers, which can be used not only in the diagnosis, but also in the prognosis of these patients. It should be noted that the study of microRNAs allows us to understand the underlying pathophysiology of genetic alterations that alter the normal process of cell differentiation and proliferation.

## 5. Conclusions

**Figure 1:**
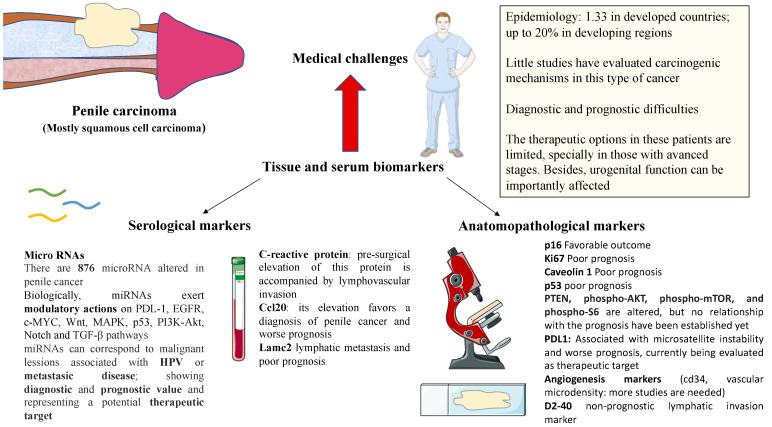
An overview of the medical challenges and potential uses of tissue and serum biomarkers. All authors made substantial contributions to the collection, sorting, image processing, editing, writing, proofreading, and revision of the manuscript and read and approved the final manuscript. Data authentication is not applicable.

Penile carcinoma is a rare cause of malignant urological neoplasia in developed countries, but has a high prevalence in developing countries. Regardless of its frequency, it is a diagnostic challenge with a multidisciplinary approach and, in many cases, its treatment causes a significant alteration in the functionality of the male reproductive system and therefore affects the quality of life of these patients. This type of review helps to provide an updated account of the situation, whilst systematic reviews such as Crocetto [67] are also very valuable. In addition, it should be noted that chemotherapy treatment in the advanced stages, in addition to being aggressive, does not have adequate response rates among patients with advanced disease. As summarized in Figure 1, different markers have been studied in penile cancer in recent years, although more effort is still required to decipher the potential clinical implications of each marker. Table 1 summarizes the current knowledge regarding this point. Although various molecular markers have been described in different urological neoplasms in recent years, this has not occurred in penile carcinoma, and practically no targeted therapy has been developed in these patients. This is why immunotherapeutic drugs are currently being evaluated in different clinical trials in combination with chemotherapy regimens to improve the prognosis of patients with advanced disease. Therefore, the future objective of the management of penile carcinoma in its different histological manifestations in terms of gene expression is based on improving early detection, identifying new molecular pathways that are candidates for targeted therapies, and describing new molecular markers in relation to prognosis based on the current lines of research in oncology.

## Figures and Tables

**Table 1:** Main markers involved and their utility.

Biomarker	Type	Utility	Reference
p16	Immunohistochemical	Better prognosis in patients with elevated expression	[8,35]
Ki67	Immunohistochemical	Higher expression is accompanied by a worse prognosis	[37]
Caveolin 1	Immunohistochemical	Higher expression is accompanied by a worse prognosis	[38]
p53	Immunohistochemical	Higher expression is accompanied by a worse prognosis	[39,40]
PDL-1	Immunohistochemical	Higher expression is accompanied by a worse prognosis; possible therapeutic target in penile carcinoma (NCT04224740)	[42]
D2-40	Density of lymphatic vessels. Immunohistochemistry.	High intratumoral lymphatic density major 2 had a sensitivity of 83.3% and a specificity of 78% in predicting lymphatic metastasis invasion.	[48]
CRP, albumin, and haptoglobin	Serological	No utility was found when used as serological markers in penile carcinoma.	[53]
CRP	Serological	Preoperative elevation of CRP is accompanied by lymphovascular invasion.	[54]
CCL20 (chemokine C-C ligands)	Serological	Preoperative serological levels were elevated compared to healthy controls: ROC of 0.855, sensitivity of 72.4%, and specificity of 93.5%.	[55]
laminin gamma 2 (LAMC2)	Serological	Serological elevation is accompanied by a greater presence of lymphatic metastases and worse survival.	[56]
miR-211-5p y miR-181d-5p	microRNA	Association with HPV-associated malignant lesions such as basaloid and verrucous carcinomas	[61]
Infraexpresión de miR-137 y miR-328-3p	microRNA	Association with metastatic disease	[61]
miR 138-5p	microRNA	Association with PDL1 expression in patients with carcinoma of the penis	[61]
miR-1, miR-101, and miR-204	microRNA	Underexpression is associated with lymphovascular and metastatic invasion in addition to presenting a worse prognosis.	[62]
miR-223-3p, miR-107, and miR-21-5p	microRNA	More frequent in carcinoma of the penis compared to non-tumorous tissue	[63]
miR-223-3p	microRNA	Overexpression is associated with a worse prognosis.	[63]
miR-145	microRNA	Underexpression is associated with perineural invasion.	[64]
miR-31-5p, miR-224-5p, and miR-223-3p	microRNA	ROC curves 0.861, 0.739, and 0.733, respectively, for differentiating penile carcinoma from non-tumorous tissue	[65]

## Data Availability

Not applicable.
